# Cerebrovascular Reactivity Following Spinal Cord Injury

**DOI:** 10.46292/sci23-00068

**Published:** 2024-05-23

**Authors:** Alexander Mark Weber, Tom E. Nightingale, Michael Jarrett, Amanda H. X. Lee, Olivia Lauren Campbell, Matthias Walter, Samuel J. E. Lucas, Aaron Phillips, Alexander Rauscher, Andrei V. Krassioukov

**Affiliations:** 1 Division of Neurology, Department of Pediatrics, University of British Columbia, Vancouver, BC, Canada; 2 BC Children's Hospital Research Institute, Vancouver, BC, Canada; 3 School of Biomedical Engineering, University of British Columbia, British Columbia, Canada; 4 Department of Neuroscience, University of British Columbia, Vancouver, BC, Canada; 5 School of Sport, Exercise and Rehabilitation Sciences, University of Birmingham, Birmingham, UK; 6 Centre for Trauma Sciences Research, University of Birmingham, Edgbaston, Birmingham, UK; 7 International Collaboration on Repair Discoveries (ICORD), University of British Columbia, Vancouver, Canada; 8 MRI Research Centre, University of British Columbia, Vancouver, Canada; 9 Department of Urology, University Hospital Basel, University of Basel, Basel, Switzerland; 10 Centre for Human Brain Health, University of Birmingham, UK; 11 Department of Physiology and Pharmacology, Cumming School of Medicine, University of Calgary, Calgary, Alberta, Canada; 12 Department of Clinical Neurosciences, Hotchkiss Brain Institute, Cumming School of Medicine, University of Calgary, Calgary, Alberta, Canada; 13 Department of Cardiac Sciences, Libin Cardiovascular Institute, Cumming School of Medicine, University of Calgary, Calgary, Alberta, Canada; 14 RestoreNetwork, Hotchkiss Brain Institute, Libin Cardiovascular Institute, McCaig Institute for Bone and Joint Health, Cumming School of Medicine, University of Calgary, Calgary, Alberta, Canada; 15 Department of Astronomy and Physics, University of British Columbia, Vancouver, BC, Canada; 16 G.F. Strong Rehabilitation Centre, Vancouver, BC, Canada; 17 Division of Physical Medicine and Rehabilitation, Faculty of Medicine, University of British Columbia, Vancouver, BC, Canada

**Keywords:** blood pressure, functional magnetic resonance imaging, spinal cord injuries, steady state cerebrovascular reactivity, tau

## Abstract

**Background:**

Spinal cord injuries (SCI) often result in cardiovascular issues, increasing the risk of stroke and cognitive deficits.

**Objectives:**

This study assessed cerebrovascular reactivity (CVR) using functional magnetic resonance imaging (fMRI) during a hypercapnic challenge in SCI participants compared to noninjured controls.

**Methods:**

Fourteen participants were analyzed (*n* = 8 with SCI [unless otherwise noted], median age = 44 years; *n* = 6 controls, median age = 33 years). CVR was calculated through fMRI signal changes.

**Results:**

The results showed a longer CVR component (tau) in the grey matter of SCI participants (*n* = 7) compared to controls (median difference = 3.0 s; *p* < .05). Time since injury (TSI) correlated negatively with steady-state CVR in the grey matter and brainstem of SCI participants (*R*_S_ = −0.81, *p* = .014; *R*_S_ = −0.84, *p* = .009, respectively). Lower steady-state CVR in the brainstem of the SCI group (*n* = 7) correlated with lower diastolic blood pressure (*R*_S_ = 0.76, *p* = .046). Higher frequency of hypotensive episodes (*n* = 7) was linked to lower CVR outcomes in the grey matter (*R*_S_ = −0.86, *p* = .014) and brainstem (*R*_S_ = −0.89, *p* = .007).

**Conclusion:**

Preliminary findings suggest a difference in the dynamic CVR component, tau, between the SCI and noninjured control groups, potentially explaining the higher cerebrovascular health burden in SCI individuals. Exploratory associations indicate that longer TSI, lower diastolic blood pressure, and more hypotensive episodes may lead to poorer CVR outcomes. However, further research is necessary to establish causality and support these observations.

## Introduction

Large population studies have indicated an increased incidence of cerebrovascular disease, such as stroke, in individuals with spinal cord injury (SCI).^1^,^2^ A recent systematic review also indicated that up to 60% of individuals with SCI suffer from global cognitive deficits.^3^ The cerebrovascular health consequences (i.e., elevated risk of stroke and cognitive dysfunction) associated with chronic SCI are likely a result of altered autonomic cardiovascular control.^4^,^5^ Indeed, an SCI at or above the sixth thoracic spinal segment (≥T6) can disrupt supraspinal sympathetic control to the heart (preganglionic neurons exiting between T1 and T5 spinal segments) and vascular tone of blood vessels in the trunk and lower extremities (preganglionic neurons exiting between T1 and L2 spinal segments). Consequently, debilitating cardiovascular impairments commonly manifest in these individuals.^6^ These manifestations include resting hypotension, a drop in blood pressure (BP) in response to an orthostatic challenge (orthostatic hypotension [OH]), and episodes of transient hypertension (autonomic dysreflexia [AD]), which is a reflex response to noxious or nonnoxious afferent stimuli from below the level of injury, such as a full urinary bladder, constipation, or sexual stimulation.^6^,^7^ Cerebral autoregulation (CA) ensures that cerebral blood flow (CBF) is maintained despite changes in perfusion pressure brought about by transient fluctuations in arterial BP,^8^ although maintained changes in BP can alter cerebral perfusion.^9^ Single episodes of extremely high BP can result in acute cerebrovascular consequences,^10^,^11^ and preclinical SCI models have demonstrated structural and functional maladaptations in the cerebrovasculature with long-term exposure to repetitive episodes of AD.^12^,^13^ Although chronic hypertension has been highlighted as one of the most prevalent risk factors for stroke^14^ and vascular cognitive impairment in the general population,^15^ less is known regarding the cerebrovascular consequences of transient and repetitive BP fluctuations experienced by humans with SCI.

One major factor shown to predict the risk of ipsilateral stroke,^16^ and associated with cognitive performance,^17^ is impaired cerebrovascular reactivity (CVR). CVR is now frequently being used as a measure of general brain health, with reduced CVR also being linked to chronic hypertension, dementia, and mild cognitive impairment in the general population.^18^,^19^ CVR represents the capacity of brain parenchyma to change CBF in response to a vasoactive stimulus (e.g., carbon dioxide [CO_2_]).^20^ A number of studies have looked at CVR responses to a vasoactive stimulus, specifically in people with SCI.^21^-^23^ However, conclusions around CVR responses in this population remain equivocal. This is due to different combinations of vasoactive stimuli and neuroimaging modalities being used across these studies, meaning the prior findings are difficult to compare. There is undoubtedly variability in the specificity, sensitivity, and spatial and temporal resolution of differing imaging strategies, with a recent study indicating no clear relationship between transcranial Doppler (TCD) and functional magnetic resonance imaging (fMRI) derived CVR measures.^24^ However, the variability of the vasoactive stimulus used in the literature (e.g., hypo- or hypercapnia or hypoxia) or the lack of repeatability in the vasodilatory effect even within a single method is of a bigger concern.^18^,^25^,^26^ It has been suggested that CO_2_ is the most suitable vasoactive stimulus,^18^ with increased CBF in response to hypercapnia being mediated by extracellular H+ through H+ sensors expressed on endothelial cells.^27^ However, a number of methods can be used to promote hypercapnia, including breath-holding, fixed inspired CO_2_, rebreathing, dynamic end-tidal forcing, or prospective end-tidal targeting, each with specific strengths and weaknesses. CVR can be assessed using a computer-controlled, MRI-compatible gas delivery system,^18^,^28^ which provides more precise control over the induced changes in arterial CO_2_ content and therefore CBF. Drawing conclusions across studies that have investigated CVR can be complicated by how the data are processed, as well as how they are acquired.

Perhaps another reason that previous CVR studies have reported equivocal findings in the SCI population is due to the fact that CVR on its own may be too broad of a metric. Indeed, CVR can be further parsed into two metrics: the response time (tau), which is the *rate* of CBF increase in response to the vasodilatory stimulus, and a static component termed steady-state CVR (ssCVR), which can be thought of as the CVR corrected for the response time (tau; see Discussion for provisos on this topic). In other words, tau represents the temporal component of CVR and ssCVR represents the time-independent component. In patients with mild dementia and Alzheimer's disease, for example, CVR response times have been found to be consistently longer than in age-matched participants.^29^-^31^ No previous trials in the SCI population have reported CVR function in terms of both steady state and response times.

We hypothesized that individuals with SCI would have reduced CVR measures compared to age- and sex-matched noninjured controls. We also aim to explore associations between free-living BP outcomes (i.e., frequency of hypotensive or AD events) and injury characteristics (i.e., time since injury [TSI] and neurological level of injury [NLI]) with novel CVR metrics in individuals with SCI. Clarifying this question will aid the understanding of the mechanisms underlying changes in cerebrovascular health following SCI and possibly contribute to the development of new rehabilitation therapies in the future.

## Materials and Methods

### Study participants

Participants living with SCI were recruited from the Greater Vancouver area, as were age- and sex-matched noninjured controls. Detailed inclusion and exclusion criteria are listed in **Table 1**. All participants provided written informed consent prior to participation in the study, which was approved by the University of British Columbia Clinical Research Ethics Board (H16-01458). Data were collected between June 2018 and February 2020. The NLI and severity of SCI (i.e., American Spinal Injury Association Impairment Scale [AIS] grade) were assessed by a board-certified physiatrist in accordance with the International Standards for Neurological Classification of Spinal Cord Injury (revised 2011).^32^

**Table 1:** Participant inclusion and exclusion criteria

Inclusion criteria	Exclusion criteria
*SCI participants* Chronic (>1-year post injury) traumatic, motor-complete SCI (AIS A or B) between C4-T6 spinal segments Documented presence of AD *All participants* Male or female 19-65 years of age Willing and able to comply with all clinic visits and study-related procedures	*All participants* History of any of the following medical conditions: untreated cardiovascular disease type 1 diabetes untreated type 2 diabetes obesity severe brain injury untreated depression Participants who have a non-MRI safe intrauterine device in place Women intending to become pregnant or currently pregnant Body mass >158 kg (maximum acceptable weight for the MR scanner) Claustrophobia Severe spasticity Standard exclusion criteria related to MR scanner (e.g., metal implants, cardiac pacemaker, shrapnel) and head circumference >63.5 cm or distance from back of head to tip of nose >24 cm (maximum possible size of a participant's head to fit into the MR head coil with face mask fitted)

*Note:* AD = autonomic dysreflexia; AIS = American Spinal Injury Association Impairment Scale; C = cervical; MR = magnetic resonance; MRI = magnetic resonance imaging; SCI = spinal cord injury; T = thoracic.

### Measuring cerebrovascular reactivity

**Figure 1:**
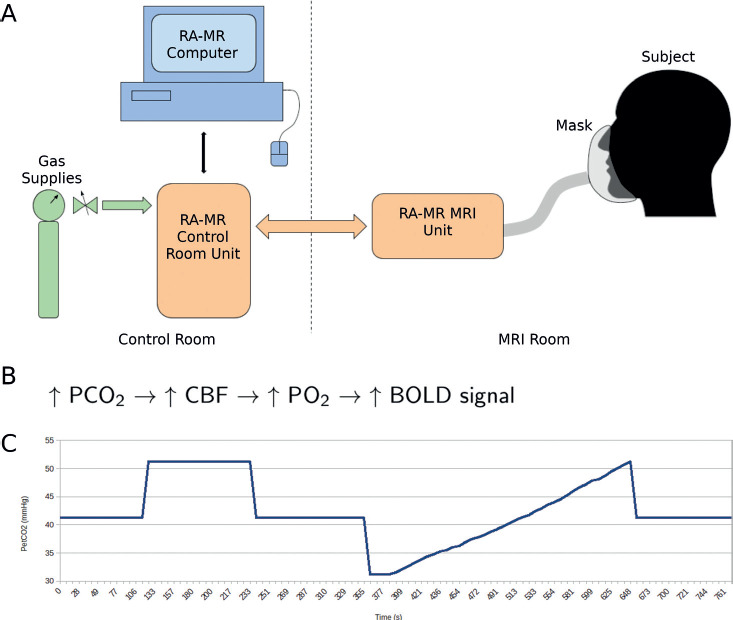
Respiract (RA-MR^TR^) and cerebrovascular reactivity (CVR) schematic diagram. (A) Provides a diagram of the basic components of the RA-MR system. The participant is breathing through a mask that has gas being detected (carbon dioxide [CO_2_] and oxygen [O_2_]) and administered by the RA-MR MRI Unit. This unit in turn receives gas from the RA-MR Control Room Unit (Gas A: 10% O_2_, 90% N_2_; Gas B: 10% O_2_, 90% CO_2_; and Gas C: 100% O_2_), which is connected directly to the gas supplies and the RA-MR computer. (B) The basic sequence of events behind hypercapnia and the increase in functional magnetic resonance imaging (fMRI) signal. (C) Target end-tidal partial pressure of carbon dioxide (P_ET_CO_2_) schematic over the course of the cerebrovascular reactivity (CVR) fMRI scan. CBF = cerebral blood flow; PCO_2_= partial pressure of CO_2_; PO_2_= partial pressure of oxygen.

In the brain, the detection of oxygen delivery needs is partly controlled by the presence of CO_2_. Increased partial pressure of CO_2_ (PCO_2_; also known as hypercapnia) will result in a dilation of the cerebral resistance vessels, which in turn results in increased CBF and PO_2_ within the cerebral tissue.^33^-^35^ Tightly controlled delivery of CO_2_ (and therefore CBF) can be accomplished using a device such as Thornhill's Respiract Gas Control System (**Figure 1A**). Given the hypercapnic-induced changes in tissue perfusion, over and above resting metabolism, arterial and venous oxygenation are elevated during a CO_2_ gas challenge. The resultant change in venous oxygenation can be measured using the blood oxygen level dependent (BOLD) effect seen in fMRI, that is, the delivery of oxyhemoglobin (HbO), a diamagnetic molecule, resulting in greater signal detection than deoxyhemoglobin (dHb), a paramagnetic molecule (**Figure 1B**). For the purposes of this study, CVR is thus defined and calculated as the percent change in BOLD signal per change in end-tidal partial pressure of CO_2_ (P_ET_CO_2_) and expressed as %ΔBOLD/mmHg.

### MRI acquisition and hypercapnic challenge

All participants were fitted with a new, unopened, and sterile gas mask, purchased through Thornhill Medical, that was cut to fit each individual's face. 3M™ Tegaderm™ Transparent Film Dressing (1624W, frame style, 6 cm x 7 cm) was cut in half lengthwise and used to ensure a leak-proof mask fitting. All scans were acquired on a Philips 3T Achieva scanner equipped with Quasar Dual Gradients and an eight-channel SENSE head coil.

The following scans were acquired with the Respiract gas mask on. Anatomical data were acquired with a high-resolution sagittal 3D T1-weighted turbo field gradient echo (TFE) sequence for registration. The imaging parameters were repetition time (TR) = 8.1 ms, echo time (TE) = 3.5 ms, flip angle = 8 degrees, acquisition matrix = 256 x 256, field of view = 256 x 256 x 160 mm, isotopic acquired and reconstructed voxel size of 1.0 x 1.0 x 1.0 mm, SENSE factor of 2 along the left-right direction, scan time = 6m 26s. Functional data were acquired with a 2D echo planar imaging (EPI) gradient-echo fMRI sequence acquired with the hypercapnia challenge (TR = 2400 ms, TE = 30 ms, flip angle = 85 degrees, acquisition matrix = 64 x 64, field of view = 224 x 224 mm, number of slices = 41, slice thickness = 3.5 mm, slice gap = 0 mm, acquired voxel size = 3.5 x 3.5 x 3.5 mm, reconstructed voxel size = 3.5 x 3.5 x 3.5 mm, EPI factor = 35, 322 volumes, SENSE factor of 2 along the anterior-posterior direction, scan time = 13m 7s). The first four scans were dummy scans to reach steady-state magnetization.

The hypercapnia challenge was achieved by inhalation of CO_2_-enriched air using the computer-controlled gas blender (RespirAct, Thornhill Research Inc., Toronto, ON, Canada). Hypercapnia was employed by applying a target P_ET_CO_2_ while maintaining end-tidal PO_2_ constant throughout (100 mm Hg). As shown in **Figure 1C**, the targeted P_ET_CO_2_ protocol was delivered as follows: first at baseline (41 mm Hg) for 2 minutes, followed by 10 mm Hg above baseline (51 mm Hg) for 2 minutes, back to baseline for 2 minutes, and next a slow ramp that started with a decrease in 10 mm Hg below baseline that proceeds to ramp up to 10 mm Hg above baseline for 5 minutes, followed by 2 minutes back at baseline. Heart rate and O_2_ saturation (SpO_2_) were acquired on all SCI and two control participants during the CVR sequence using an Invivo Essential MRI Patient Monitor (Invivo Corp., Orlando, FL).

### Image preprocessing and CVR calculation

**Figure 2:**
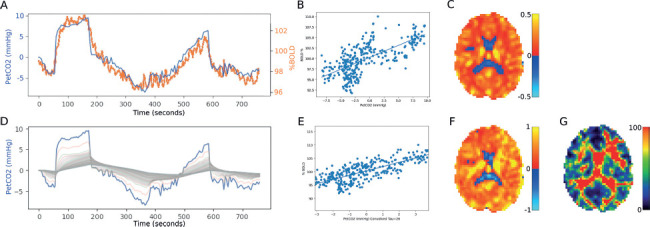
Cerebrovascular reactivity (CVR) processing. All data are from a sample SCI participant. (A) Alignment of end-tidal PCO_2_ (P_ET_CO_2_ [blue]) with the average blood oxygen level dependent (BOLD) signal (orange). (B) Linear least-squares fit of the BOLD response to the P_ET_CO_2_ stimulus on a voxel-by-voxel basis across the whole gas challenge protocol: CVR_whole_. (C) Resulting axial slice of a CVR map (in units Δ%BOLD/mmHg [percent change in BOLD signal per change in end-tidal partial pressure of CO_2_]). (D) Set of curves resulting from the convolution of P_ET_CO_2_. (E) The regression with the best fit between the convolved P_ET_CO_2_ and the BOLD signal: steady-state CVR (ssCVR). (F) Resulting axial slice of a ssCVR map. (G) Resulting axial slice of a tau map.

Structural T1-weighted image processing included a file conversion to Neuroimaging Informatics Technology Initiative (NIFTI) format, bias-field correction, spatial normalization, and segmentation using fmriprep (fmriprep_ docker-20.1.3.^36^); fmriprep uses the FMRIB (Oxford Centre for Functional Magnetic Resonance Imaging of the Brain) Software Library (FSL^37^), Advanced Normalization Tools (ANTs^38^), FreeSurfer,^39^ and Analysis of Functional NeuroImages (AFNI^40^). fMRI image preprocessing, including motion correction, slice-timing correction, alignment to T1-weighted image, and alignment to Montreal Neurological Institute (MNI) space, was performed using fmriprep. The BOLD reference was coregistered to the T1w reference using “bbregister” (FreeSurfer), which implements boundary-based registration.^41^ Coregistration was configured with six degrees of freedom. Head-motion parameters with respect to the BOLD reference (transformation matrices and six corresponding rotation and translation parameters) are estimated before any spatiotemporal filtering using “mcflirt” (FSL 5.0.9^42^). The BOLD time-series (including slice-timing correction when applied) were resampled onto their original, native space by applying the transforms to correct for head motion. These resampled BOLD time-series will be referred to as *preprocessed BOLD in original space* or just *preprocessed BOLD*. The BOLD time-series were resampled into standard space, generating a *preprocessed BOLD run in MNI152NLin2009cAsym space*. Gridded (volumetric) resamplings were performed using antsApplyTransforms (from ANTs), configured with Lanczos interpolation to minimize the smoothing effects of other kernels.^43^ Nongridded (surface) resamplings were performed using mri_vol2surf (FreeSurfer). CVR maps were calculated as follows. The recorded P_ET_CO_2_ stimulus was time shifted to the point of maximum correlation with the whole-brain average BOLD signal. CVR was then calculated as a linear, least-squares fit of the BOLD response (S) to the P_ET_CO_2_ stimulus on a voxel-by-voxel basis across the whole gas challenge protocol (**Figure 2**). To differentiate this from the other two CVR outcome measures reported, we defined this as CVR_whole_.

Tau and ssCVR voxel-wise maps were calculated as follows. First the CVR was parsed into a steady-state component (ssCVR) and a dynamic component (τ). The principles of measuring these parameters are outlined schematically in **Figure 1**.^44^ The BOLD response was modeled as the convolution of the measured P_ET_CO_2_ and an exponential decay function of the form exp(-t/τ), where t is time and τ the time constant of response. Convolutions were calculated for each voxel for values of τ between 0 and 200 in intervals of 4. The τ value for each voxel was determined by the convolution with the highest Pearson's *r* correlation. The modeled convolution for various τ values is shown in **Figure 2D**. The ssCVR values were computed using numpy.polyfit to calculate the slope of the regression between the convolved P_ET_CO_2_ with the optimal τ value and the BOLD response for each voxel. ssCVR represents the time-independent response, with the same units as CVR_whole_ (Δ%BOLD/mmHg), while τ represents the speed of the CVR response to hypercapnia. Regional mean CVR values were obtained from two regions of interest chosen a priori: the grey matter and brainstem. These region of interest (ROI) masks were obtained from the output of the fmriprep analysis.

### Free-living blood pressure monitoring

Ambulatory BP monitoring (ABPM) devices (Meditech ABPM-04, Budapest, Hungary) were used to track participants’ BP over a continuous 24 hours of daily living. This approach is reliable and well-validated for capturing free-living cardiovascular profiles in individuals with SCI.^45^ BP was measured at 30-minute intervals between 6:00 a.m. and 9:00 a.m., at 15-minute intervals between 9:00 a.m. and 9:00 p.m., and every hour between 9:00 p.m. and 6:00 a.m. Participants were also instructed to manually record BP and keep a diary to track any key events or record any signs and symptoms of BP instability (i.e., sleep and wake times, bowel movements, bladder emptying, exercise, AD or hypotensive signs and symptoms, etc.) throughout the duration of the 24-hour ABPM assessment day. Baseline BP values were established as the average of three measurements taken from the SCI participants’ nondominant arm while seated in their own wheelchair, as has been done previously.^46^-^48^ These values were subsequently used as a reference point to assess the frequency of AD events during the daytime. AD was characterized as an increase in systolic BP (SBP) >20 mm Hg above measured baseline SBP, as per the International Standards to Document Remaining Autonomic Function after Spinal Cord Injury.^49^ AD events during the nighttime were determined using a baseline nighttime SBP, which was calculated as the average of the first three automatic BP measurements when the participant was asleep. The definition of a hypotensive event is somewhat ambiguous; in the context of orthostatic hypotension, a baseline is often used as the benchmark. We have characterized a hypotensive event using the following criteria that has previously been applied in the SCI population: SBP ≤100 mm Hg and diastolic BP (DBP) ≤70 mm Hg.^50^ Hypotensive events were not calculated during the nighttime because of the potential for a nocturnal dip in BP. Average daytime SBP and DBP were also calculated from readings where the participant self-reported as being awake. Twenty- four-hour ABPM data were only considered valid if there were >65% successful automatic readings during the measurement period.

### Statistical analysis

All statistics were performed using R version 4.1.2 (“Bird Hippie”). Due to the small sample size, all statistics were performed and are reported using nonparametric methods. Results are reported using median and interquartile range (IQR) in parentheses. Mann-Whitney *U* tests were conducted to analyze differences between the two cohorts (age, CVR measures, etc.). As an exploratory analysis, Spearman's rank correlation coefficients were performed for the SCI cohort only to explore associations between demographic and injury characteristics (e.g., age, TSI, and NLI), physiological outcomes measured during the MR scan (e.g., average heart rate [HR] and SpO_2_ during the hypercapnic challenge) and 24-hour ABPM outcomes (e.g., daytime mean SBP and DBP, daily episodes of hypotension and AD), with CVR measures (e.g., CVR_whole_, ssCVR, and tau) of the grey matter and brainstem. These associations will be presented as a correlation matrix. NLI was modified into an ascending, continuous numeric variable for analysis (i.e., C4 = 1, C5 = 2, C6 = 3, etc.). Significance was set at an alpha level of 5%, uncorrected.

## Results

**Figure 3:**
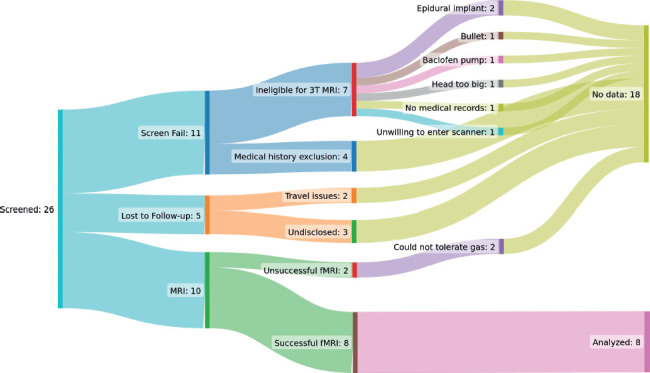
SCI participant recruitment flow diagram. Of the 26 SCI participants who were screened, 10 were able to be scanned in the MRI, while the remaining 16 either failed the screening (11) or were lost to follow-up (5). The diagram further breaks down the reasons for failure. Of the 10 participants who were scanned, 8 were able to tolerate breathing the gas mixture. Of these 8 participants with functional magnetic resonance imaging (fMRI) data, all 8 were included for the exploratory associations with participant demographics and injury characteristics; 7 were included for exploratory associations with 24-hour ambulatory blood pressure monitoring (ABPM) outcomes as one participant had less than 65% weartime/successful readings; and 7 were included for cohort comparison with control participants as one participant did not have an appropriate age/sex match. Please see the eTable for a breakdown of which participants were included for which analysis.

Ten participants with cervical or upper thoracic motor-complete SCI were enrolled in this study. We encountered many obstacles when attempting to send individuals with SCI to the scanner (see **Figure 3** and Limitations). Two participants experienced a panic attack from breathing the CO_2_ rich gas, in which case no fMRI scans were acquired, and they were excluded from our analysis. Eventually COVID-19 restrictions and scanner decommissioning put an end to data collection. Because of this, we resorted to using noninjured participants (*n* = 2) from the development scans in our control cohort in order to obtain as close to age- and sex-matched controls as possible. A suitable noninjured control close in age to participant ID#8 was not recruited in time. As such, this participant was removed from the cohort comparison but was retained in the correlation analysis. Consequently, successful fMRI scans were obtained from eight participants with SCI and used in the correlation analysis. For the cohort comparison, fMRI scans from seven male participants with SCI, with a median age of 42 (19) years and an age range of 21 to 53 years, and six noninjured controls (all males), with a median age of 33 (11) years and an age range of 24 to 42 years, were used in the analysis. No significant difference was found between the age of the two groups (*p* = .315). **Table 2** provides a summary of the cohorts for a range of outcome measures. Individual participant data are also provided in **Table 3**. The 24-hour ABPM was well tolerated, with an 89% (17%) success rating for automated measurements and total measurements being 63 (11) across all participants. Daytime SBP was significantly higher for noninjured control participants compared to participants with SCI [127 (6) vs. 107 (21) mm Hg; *p* = .01]. Participants with SCI had significantly (*p* < .042) more hypotensive episodes and events classified as AD compared to noninjured controls (**Table 2**).

**Table 2:** Participant characteristics, physiological variables measured in the MR scanner, and 24-hour ambulatory blood pressure outcomes summarized for the cohort comparison analysis

Outcome	SCI (C4-T5) (*n* = 7)	Noninjured age- matched controls (*n* = 6)	*p* value
**Participant demographics^a^**			
Age, years	42 (19)	33 (11)	.315
AIS, A/B	4 (57%)/3 (43%)	N/A	—
TSI, years	13 (20)	N/A	—
Weight, kg	72.2 (20.1)	75.9 (6.8)	.257
**Physiological variables**	**measured in MR scanner**	**during CVR^a^^b^**	
HR, bpm	61 (12)	64 (N/A)	1.000
SpO_2_, %	98 (3)	98 (N/A)	.766
**24-hour ABPM^c^**			
SBP, mm Hg	107 (21)	127(6)	.011*
DBP, mm Hg	69 (18)	71 (10)	.391
Total AD events	9 (12)	3 (3)	.037*
Daytime hypotensive	6 (26)	0 (1)	.042*
events			

*Note:* Values given as median (IQR) or *n* (%). ABPM = ambulatory blood pressure monitoring; AD = autonomic dysreflexia; AIS = American Spinal Injury Impairment Scale; bpm = beats per minute; CVR = cerebrovascular reactivity; DBP = diastolic blood pressure; HR = heart rate; MR = magnetic resonance; N/A = not applicable; NLI = neurological level of injury; SpO2 = oxygen saturation level; SBP = systolic blood pressure; TSI = time since injury.

* Significant at *p* < .05.

a *n* = 4 noninjured control participants missing weight, physiological variables measured in MR scanner, and 24-hour ABPM for the two development scan participants.

b *n* = 2 for physiological data measured in MR scanner during cerebrovascular reactivity (CVR) for noninjured age matched control participants due to device malfunction. Hence IQR is N/A.

c *n* = 6 for 24-hour ABPM data for SCI participants due to insufficient wear time (<65%) for one participant.

**Table 3:** Individual demographic and injury characteristics, 24-hour ABPM, and fMRI data for all SCI participants

ID	Age, years	TSI, years	NLI	AIS	HR, bpm	SpO_2_, %	SBP, mmHg	DBP, mmHg	Total AD events	Total hypotens events	GM CVR _whole_	GM ssCVR	GM tau	BS CVR_whole_	BS ssCVR	BS tau
1	50-55	0-5	T5	A	74	98	115	76	16	0	0.33	0.45	13.6	0.25	0.50	16.53
2	30-35	16-20	C5	B	58	99	107	60	8	7	0.28	0.38	11.76	0.26	0.34	13.39
3	50-55	0-5	C5	B	56	95	98	61	8	21	0.27	0.39	10.73	0.24	0.41	12.21
4	30-35	10-15	T2	A	68	99	115	68	2	4	0.36	0.44	15.59	0.31	0.48	16.97
5	20-25	0-5	T3	A	53	100	n/a	n/a	n/a	n/a	0.34	0.45	10.14	0.30	0.43	7.06
6	40-45	20-25	C4	A	61	98	84	54	10	53	0.25	0.34	13.86	0.23	0.28	15.21
7	46-50	20-25	C7	B	61	96	90	54	25	5	0.29	0.39	13.81	0.24	0.35	13.69
8^a^	60-65	40-45	T4	A	59	99	113	60	5	14	0.24	0.37	12.11	0.20	0.26	9.62

*Note:* ABPM = ambulatory blood pressure monitoring; AD = autonomic dysreflexia; AIS = American Spinal Injury Impairment Scale; bpm = beats per minute; BS = brain stem; CVR = cerebrovascular reactivity; DBP = diastolic blood pressure; fMRI = functional magnetic resonance imaging; GM = grey matter; HR = heart rate; ID = anonymized participant ID for publication; NLI = neurological level of injury; SBP = systolic blood pressure; SpO_2_, = oxygen saturation; ssCVR = steady-state CVR; TSI = time since injury; Total hypotens events = total daytime hypotensive events.

a Participant ID#8 was not included when performing statistics comparing differences with control participants as there was no age-matched control available.

### Differences in CVR metrics between SCI and noninjured control participants

**Figure 4:**
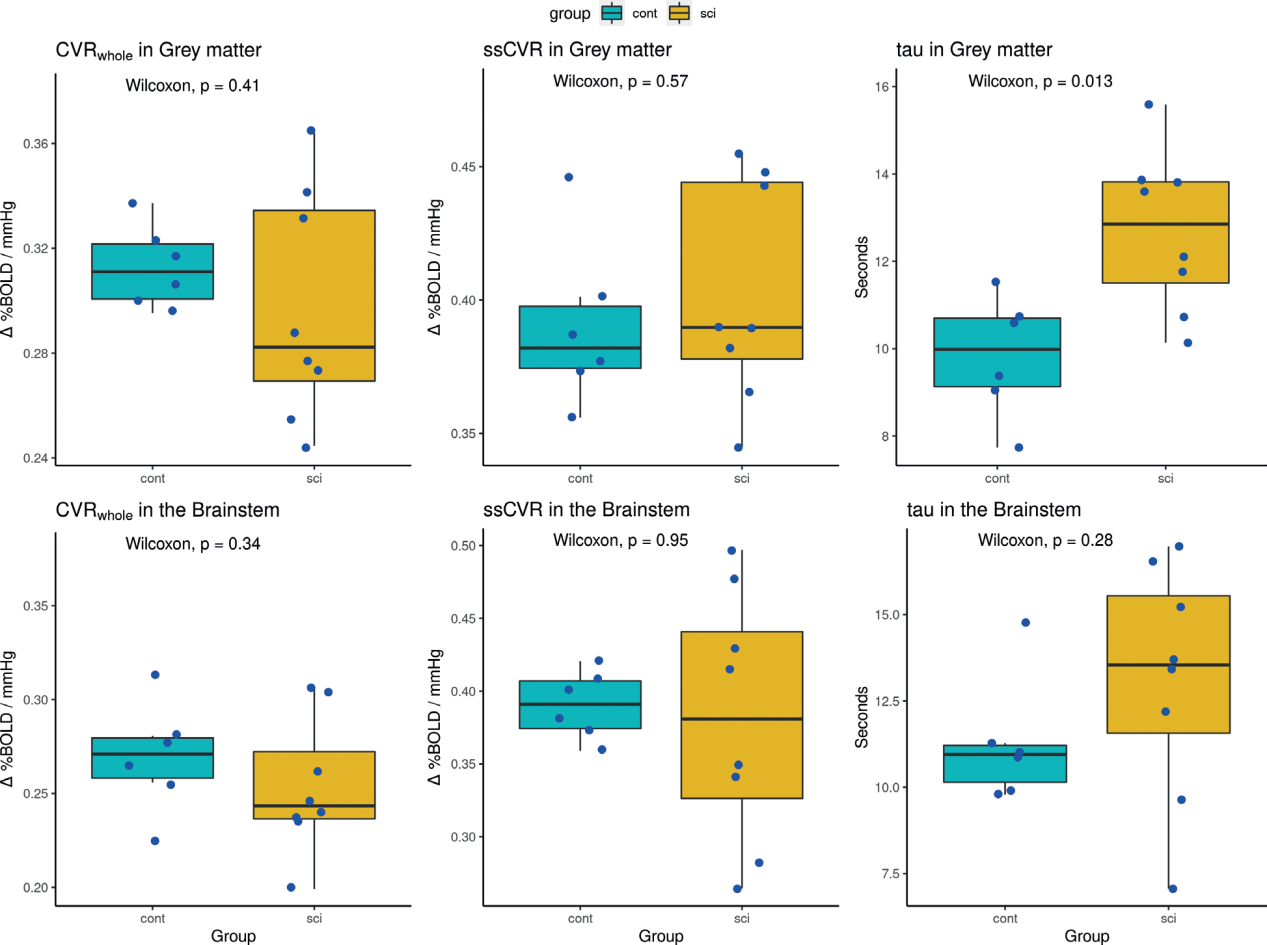
Boxplots comparing group results. Noninjured controls are shown in cyan color, and SCI participants are shown in yellow. Exact values of data with demographic and injury characteristics can be found in Table 3. For example, the SCI participant with the high tau value in the grey matter is young (31 years) with a T2 injury. They only experienced two episodes of autonomic dysreflexia and four episodes of hypotension (i.e., less blood pressure instability relative to the rest of the cohort). Δ%BOLD/mmHg = percent change in BOLD signal per change in end-tidal partial pressure of CO_2_; cont = control; CVR_whole_ = linear least-squares fit of the BOLD response to the target end-tidal PCO_2_ stimulus on a voxel-by-voxel basis across the whole gas challenge protocol; sci = spinal cord injury; ssCVR = steady-state cerebrovascular reactivity.

Group differences in the grey matter and brainstem were explored for CVR_whole_, ssCVR, and tau. A statistically significant difference in tau in the grey matter between the SCI and noninjured cohorts was found, with a ∼25% longer tau found in the SCI cohort (median of the difference = 3.00 seconds; *p* < .05). Boxplots are shown in **Figure 4**.

### Associations between injury characteristics and free living BP variability with fMRI metrics in the SCI cohort

Brainstem CVR_whole_ (R_S_ = −0.73, *p* = .040) and ssCVR in the grey matter (R_S_ = −0.81, *p* = .014) and brainstem (R_S_ = −0.84, *p* = .009) all showed significant negative correlations with TSI (**Table 4**). CVR response time (tau) in the grey matter and brainstem were found to have a statistically significant positive correlation with HR measured in the scanner during the hypercapnic stimulus (R_S_ = 0.85, *p* = .007; R_S_ = 0.90, *p* = .002, respectively).

**Table 4:** Correlation matrix

	Age	TSI	NLI	HR	SpO_2_, %	GM CVR_whole_	GM ssCVR	GM tau	BS CVR_whole_	BS ssCVR	BS tau
**Age**		0.42	0.17	0.13	-0.59	-0.69	-0.43	-0.05	**-0.76**	0.33	-0.12
**TSI**			-0.28	0.18	-0.15	-0.68	**-0.81**	0.44	**-0.73**	**-0.84**	0.04
**NLI**				0.27	0.37	0.35	0.59	-0.11	0.14	0.43	-0.06
**HR**					-0.24	0.23	0.05	**0.85**	-0.04	0.31	**0.90**
**SpO_2_, %**						| 0.32	0.25	-0.20	0.45	0.00	-0.28
**SBP (*n*=7)**	-0.04	-0.45	**0.82**	0.44	0.54	0.52	0.63	0.05	0.54	0.52	0.31
**DBP (*n*=7)**	0.04	**-0.81**	0.61	0.33	0.14	0.55	0.71	-0.11	0.62	**0.76**	0.35
**Total AD events (*n*=7)**	0.14	-0.07	-0.09	0.24	-0.64	0.09	0.16	0.02	-0.25	0.14	0.13
**Total daytime hypotensive events (*n*=7)**	0.25	0.47	-0.76	-0.68	-0.22	**-0.86**	**-0.89**	-0.29	-0.61	-0.71	-0.57

*Note: n* = 8 unless otherwise noted. Participant ID#5 wore the 24-hour ambulatory blood pressure monitor (ABPM) on two occasions; on both occasions there was an insufficient amount of successful automatic readings (<65%). Values are Spearman's rank correlation coefficients (Rs). Bold values have *p* < .05. AD = autonomic dysreflexia; BS = brainstem; CVR_whole_ = linear least-squares fit of the blood oxygen level dependent response to the target end-tidal PCO_2_ stimulus on a voxel-by-voxel basis across the whole gas challenge protocol; DBP = diastolic blood pressure; GM = grey matter; HR = heart rate; NLI = neurological level of injury; SBP = systolic blood pressure; SpO_2_ = oxygen saturation; ssCVR = steady-state cerebrovascular reactivity; TSI = time since injury.

Daytime SBP was found to be correlated with NLI (R_S_ = 0.82, *p* = .024) but was not found to be correlated with age, HR, TSI, SpO_2_, or any CVR measure. In the grey matter, daytime DBP was not statistically significantly correlated with CVR_whole_, ssCVR, or tau. In the brainstem, daytime DBP was correlated with ssCVR (R_S_ = 0.76, *p* = .046) but not CVR_whole_ or tau. Daytime DBP was found to also be correlated with TSI (R_S_ = −0.81, *p* = .005) but not with age, HR, NLI, or SpO_2_. Total AD episodes were not statistically significantly correlated with any measures. However, hypotensive episodes were found to be negatively correlated with NLI (R_S_ = −0.76, *p* = .049), CVR_whole_ in the grey matter (R_S_ = −0.86, *p* = .014), and ssCVR in the grey matter (R_s_ = −0.89, *p* = .007).

## Discussion

This exploratory study is the first of its kind, in which CVR responses during the delivery of a consistent and repeatable PCO_2_ stimulus were collected using fMRI BOLD and compared between noninjured controls and participants with SCI. As people with SCI face up to four times the risk of ischemic and hemorrhagic stroke, along with up to 60% of those injured becoming cognitively impaired,^2^,^3^ it is clear this population is at risk of some form of cerebral health dysfunction. We set out to measure whole-brain cerebrovascular health using multiple CVR measures: CVR_whole_, ssCVR, and tau (CVR response time). The new findings from this preliminary study are that (1) whole-brain cerebrovascular reactivity to CO_2_ (i.e., CVR_whole_) is unchanged in participants with SCI, (2) CVR response times (tau) in participants with SCI were ∼25% longer in grey matter than in noninjured participants, and (3) TSI and the frequency of hypotensive episodes were negatively correlated with CVR outcomes. Although causation cannot be inferred, collectively, these preliminary associations indicate hypotension (and ultimately brain hypoperfusion) is linked with impaired CVR responses in individuals with SCI.

### Previous cerebrovascular studies in SCI

Phillips et al.^13^ recently developed a preclinical rodent model of AD after SCI. The authors induced repetitive transient hypertension over a 4-week period in a group of rodents (*n* = 25) after a T3 spinal transection to mimic AD (T3-SCI+AD). Compared to a control group of SCI rats (T3-SCI), the T3- SCI+AD group had impaired cerebrovascular health, including reduced cerebrovascular endothelial function and perivascular sympathetic cerebrovascular innervation. However, they found no differences in CBF between the groups as measured using MRI (arterial spin labelling) on a 7T scanner. There was also no evidence of cerebrovascular inward remodeling, leading the authors to speculate that repetitive transient hypertension is a unique representation of impaired cerebrovascular health compared to chronic steady-state hypertension in noninjured individuals.

There have only been a handful of studies that have looked at cerebrovascular function in humans with chronic SCI. The results are mixed, with findings showing that high-thoracic and cervical SCI can impair, can have no effect, or can even improve cerebral autoregulation.^5^ In 1972, one of the first studies to look at cerebrovascular function after SCI used inhaled ^133^Xe along with hyperventilation (hypocapnia) in 12 participants with cervical injuries (C5-C7) compared to five participants with thoracic (T2-T4) injuries as controls.^21^ They found no difference in hypercapnic CBF reactivity between the two cohorts. However, they did find a lack of cerebrovascular responses to hypocapnia in individuals with cervical SCI, with responses in individuals with thoracic injuries being intact. Four years later, using the same technique, Nanda et al.^22^ found no cerebrovascular response differences to hypocapnia between individuals with cervical injuries (*n* = 8) and noninjured controls (and one subject with a T2-3 lesion; *n* = 13). How to reconcile the discrepancy between these two studies remained unclear. Three decades later, Wilson et al.^23^ used TCD (for blood velocity in middle cerebral artery) and near-infrared spectroscopy (for activation-dependent information in the prefrontal cortex) to look at hypercapnia and hypocapnia CVR in six men with C5-C7 lesions and 14 noninjured men. They found no difference in either hyper- or hypocapnia between the SCI and able-bodied cohort. When looking at cerebrovascular resistance (instead of blood velocity or vascular conductance), however, they reported a 30% decrease in SCI participants in response to hypocapnia (but not hypercapnia). While the authors interpreted this result as being reflective of subtle differences in CBF modulation, Kim and Tan^5^ in their review of CVR in SCI disagreed and suggested a more likely explanation could be different arterial pressure responses to hypocapnia in individuals with high-level SCI. They reasoned that reduced lung volumes and flow rates due to the disruption of neural innervation of respiratory muscles in SCI leads to impaired mechanics of ventilation, which in turn may impact arterial pressure during hyperventilation. Thus, according to the available literature, whether SCI impairs cerebrovascular reactivity remains unanswered and has previously been limited by the assessment methods (e.g., more variability when not using end-tidal CO_2_ clamping systems such as the RespiractTM or a lack of spatiotemporal information from TCD) and study designs used (e.g., using individuals with upper thoracic injuries who may still experience unstable BP as controls).

### What our preliminary findings show

Previous SCI studies have not reported a difference in CVR in response to hypercapnia between SCI and noninjured cohorts. Our study also did not find a difference using CVR on its own (i.e., CVR_whole_). However, by breaking the CVR measure into its steady-state and active components, we reveal an important difference between the cohorts in the response time (tau) and significant correlations between TSI and the steady-state component (ssCVR). These findings may hold the key to explaining the equivocal CVR findings reported in previous studies and highlight the consequences of living with an SCI for a longer time.

In all participants, the BOLD signal achieved a better fit to the convolved P_ET_CO_2_ than to the actual P_ET_CO_2_; an example can be seen in **Figure 2**. This is supported by the higher Spearman's rank correlation coefficients of TSI and ssCVR in the grey matter and brainstem (-0.81 and −0.84, respectively) versus TSI and CVR_whole_ (-0.68 and −0.73, respectively), suggesting that ssCVR is more sensitive than CVR_whole_. While no differences were found between ssCVR in the two cohorts, these aforementioned associations indicate the more chronic the injury, the more reduced a participant's ssCVR.

As CVR is by definition a dynamic measure, it logically follows that its speed of response should be examined. A long response time in CVR suggests reduced cerebrovascular health and increased risk of ischemic damage, Alzheimer's disease, or stroke, and it has been associated with the development of white matter hyperintensities.^51^-^53^ CA is a hallmark of the mammalian brain and is needed to prevent ischemic damage at low BP and stroke at high BP.^54^ These extremes of BP are key characteristics in SCI, especially during OH^55^ and AD.^56^ CA adjusts cerebrovascular resistance to ensure that CBF is in line with metabolic needs and is itself composed of both static and dynamic components. Dynamic CA is the rapid regulation of CBF in response to altered arterial BP, whereas static CA is responsible for limiting CBF changes over gradual perfusion pressure changes. It is interesting that we report differences in the dynamic tau measure (i.e., decreased speed of vascular response) but not ssCVR between the two cohorts. Recent work suggests that the shear-mediated dilation response to a transient CO_2_ stimulus is more endothelium-dependent than the ssCVR component.^57^ These data may therefore imply impaired nitric oxide-mediated cerebrovascular function in humans with SCI.

### Suggested links with free-living blood pressure variables

DBP was found to be positively correlated with ssCVR in the brainstem and with TSI. Frequency of hypotensive events revealed widespread negative associations with NLI, CVR_whole_ (in grey matter), and ssCVR (in grey matter). Thus, a lower CVR_whole_ and ssCVR in grey matter was generally correlated with lower BP. It is possible that brain hypoperfusion as a result of low systemic BP is responsible for the lower CVR seen in these participants. Alternatively, the low BP causes vessels to dilate to maintain normal baseline perfusion in response to reduced perfusion pressure. Under these circumstances, the capacity for further dilation in response to CO_2_ can become reduced since the vessels are already dilated due to autoregulated compensation.^58^ Given that responses to a CO_2_ stimulus are sigmoidal,^59^ individuals with SCI and low BP are encroaching on the nonlinear portion of the response curve. These findings align with other research in the SCI population, whereby responses to altered transient metabolic demand (e.g., neurovascular coupling [NVC]) were manipulated by an eyes-open and eyes-closed task.^20^ Phillips et al.^20^ demonstrated an absent posterior cerebral artery blood velocity response compared to noninjured controls and that this response was normalized when BP was increased in the SCI cohort using midodrine (an alpha-1 agonist). The authors noted that this improvement in NVC was also related to improved cognitive function. Despite preclinical evidence suggesting repetitive exposure to AD results in structural and functional changes in the cerebrovasculature,^12^,^13^ total frequency of AD events in our study was found not to be correlated with any CVR measure. Looking outside of the cerebrovasculature, Currie et al.^60^ revealed no correlations with arterial stiffness (measured via carotid-to-femoral pulse wave velocity [cfPWV]) and frequency of AD events derived using 24- hour ABPM, although there were correlations with the frequency of hypotensive events. When AD and hypotensive events (total episodes of BP instability) were combined, significant positive correlations were observed with cfPWV, which is a prognostic risk factor for cardiovascular disease.^61^ A recent study raised the possibility that vascular stiffness contributes to altered cerebrovascular hemodynamics and impaired cognitive function in older adults.^62^ Although our findings suggest a stronger link between low BP and CVR outcomes, the daily fluctuations in BP experienced by this population (demonstrated in **Table 2**) likely contribute to the deterioration in cerebrovascular health.

### Considerations

Several limitations of the present study should be considered when interpreting the results. First, the sample size is small with respect to both SCI and noninjured control participants. Recruiting participants with SCI can be challenging,^63^ particularly with specific injury characteristics and hemodynamic instability. This was further compounded when it came to acquiring the necessary medical records detailing spinal hardware or other implants to ensure MR compatibility and safety when scanning at 3T. When MRI compatibility was finally acquired, we encountered further difficulties during scanning (see Results). Second, although we attempted to recruit females in this study, the data analyzed were solely from male participants. Thus, our findings should not be assumed to apply to females with SCI until further studies can be performed. There is a notable sex and gender gap in SCI research, and future CVR studies in this population should consider adopting a more sex- and gender-sensitive research design.^64^

We were unable to measure BP while participants were in the scanner and images were being acquired. An increase in BP has previously been noted during hypercapnia, with less robust increases shown in other clinical groups with disrupted autonomic cardiovascular control (e.g., multiple-system atrophy) compared to controls.^65^ Not accounting for BP changes during hypercapnia between participants and cohorts means we are unable to provide more insight into the pure “cerebrovascular” response as opposed to perfusion pressure driving the increased CBF. Despite attempts to minimize triggers for AD with gel padding, it is possible that prolonged lying might have resulted in transient elevations in BP in the SCI cohort,^66^ which may have influenced CVR responses. That being said, HR was stable during scans and participants did not exhibit bradycardia, a common physiological sign associated with AD. Body position plays a role in BP and brain perfusion in individuals with cervical and upper thoracic injuries.^67^ Therefore, it remains to be seen whether CVR responses characterized by other imaging modalities (e.g., functional near-infrared spectroscopy, TCD, or magnetoencephalography) would be more pronounced between individuals with SCI and noninjured controls if the hypercapnic stimulus was provided in the seated position. Measuring continuous, beat-by-beat BP during an 3T MR scan was historically questionable due to the lack of reliable MR safe devices. Given recent technological advancements, MR-safe BP measurement solutions are now available. Future research might want to consider using these, although their validity and reliability remain to be elucidated.

Furthermore, our description of tau may be oversimplified. It is important to note that the “tau” parameter is essentially a dispersion parameter, influenced not only by flow changes but also by drainage. This dispersion is weighted by changes in vessel responsiveness and by venous contrast due to the BOLD signal. Therefore, strictly attributing dispersion solely to changes in vessel responsiveness may not be accurate.^68^

There are other confounding factors that may explain the differences in tau between individuals with SCI and noninjured controls that were not measured or controlled for in this current study. Evidence suggests that individuals with SCI are inherently less physically active when compared to the general population,^69^ which leads to profound physical deconditioning.^70^ We are not aware of any literature linking physical activity and cardiorespiratory fitness status with this dynamic tau measure, specifically in the cerebrovasculature. However, exercise improves global brain health, including cerebrovascular function,^71^,^72^ and physical inactivity has been associated with impaired vasoreactivity in the peripheral vasculature.^73^ The prevalence of sleep-disordered breathing in the SCI population is high.^74^ Participants were not screened for obstructive sleep apnea, and sleep quality was not characterized in this current study. It has been suggested that sleep-disordered breathing may be confounding rehabilitation research^75^ and could partially underpin the increased risk of stroke in the SCI population.^76^ Recent evidence looking specifically at fMRI-derived CVR indicates the response to a hypercapnic stimulus is greater in individuals with obstructive sleep apnea relative to controls,^77^ possibly representing a compensatory mechanism that requires further investigation. Therefore, researchers may want to consider utilizing animal models of SCI to control for such confounding variables, such as physical activity status or sleep-disordered breathing, to better elucidate the mechanisms of impaired cerebrovascular health following SCI.

We wanted to share the aforementioned factors to highlight the logistical difficulties experienced with this study and key considerations for researchers when scanning this population at 3T. Collaborative research (through consortia and multicenter trials) should be encouraged to facilitate larger sample sizes, although variability between study sites and scanners will need to be accounted for in statistical analyses. Encouragingly, recent research has suggested that individual CVR testing is compatible across sites, providing that standardized respiratory stimuli and BOLD MRI scan parameters are used.^78^,^79^

## Conclusion

Our results demonstrate that the CVR response time (tau) to hypercapnia is increased with SCI in grey matter compared to noninjured participants. Despite decades of equivocal or null findings of hypercapnia CVR in SCI— a mystery given the higher incidence of cerebrovascular health problems in the SCI population— we report for the first time a possible explanation. Although CVR_whole_ appears unchanged with SCI, its dynamic and static components reveal another story: (1) CVR response time is increased (∼25%) in individuals with SCI, and (2) impaired ssCVR is associated with a greater TSI. We also noted associations between low BP and CVR outcomes. However, these preliminary findings should be viewed with caution given a lack of corresponding BP data collected during the MR scan, small sample size, the analysis of only male participants, and cross-sectional study design. Further work is necessary to support these observations. We have also provided a number of considerations for authors wishing to investigate CVR in clinical populations in the future.

## Supplementary Material


