# Supplementary material for: Cerebrovascular Reactivity Following Spinal Cord Injury
Source: Top Spinal Cord Inj Rehabil. 2024 May 23;30(2):78–95. doi: 10.46292/sci23-00068 (PMC11123610; doi:10.46292/sci23-00068)
Supplement: Supplementary file 1 [file i1537-2073-30-2-78-s01.pdf]

eTable. Participant characteristics for individuals with SCI enrolled in the trial and available outcome data

	**Participant characteristics**					**MRI sequence**		**24-hr ABPM**	**Age/sex match control**
**ID**	**Age range, years**	**Sex**	**TSI, years**	**NLI**	**AIS**	**3DT1**	**fMRI (CVR)**		
1	46-50	M	2	T5	A	Yes	Yes	Yes	Yes
2	61-65	M	45	T4	A	Yes	Yes	Yes	**No**
3	31-35	M	17	C5	B	Yes	Yes	Yes	Yes
4	46-50	F	34	T5	A	Yes	**No**	Yes	**n/a**
5	51-55	M	5	C5	B	Yes	Yes	Yes	Yes
6	31-35	M	4	C6	B	Yes	**No**	Yes	**n/a**
7	31-35	M	13	T2	A	Yes	Yes	Yes	Yes
8	41-45	M	22	C4	A	Yes	Yes	Yes	Yes
9	21-25	M	1	T3	A	Yes	Yes	**No**	Yes
10	46-50	M	22	C7	B	Yes	Yes	Yes	Yes
All SCI, median (IQR) (*n*=13)	44 (17)	9M/1F	15 (18)	5C/5T	6A/4B		*n* = 8	*n* with fMRI data = 7	*n* = 7
**Participant characteristics for cohort comparisons**									
SCI, median (IQR) (*n* = 7)	42 (16)	7M	13 (16)	4C/3T	5A/2B				
Controls, median (IQR) (*n* = 6)	33 (9)	6M	-	-	-				
**Participant characteristics for exploratory association analysis**									
SCI, median (IQR) (*n* = 8)	44 (18)	8M	15 (18)	4C/4T	5A/3B				

Note: AIS = American Spinal Injury Impairment Scale; C = cervical; CVR = cerebrovascular reactivity; F = female; fMRI = functional magnetic resonance imaging; M = male; NLI = neurological level of injury; T = thoracic; TSI = time since injury; 3DT1 = 3D T1-weighted image.

This supplementary digital material accompanies:
Top Spinal Cord Inj Rehabil 2024;30(2):78-95
© 2014 American Spinal Injury Association
www.asia-spinalinjury.org
doi: 10.46292/sci23-00068
